# Evaluation of the Effectiveness of Cinnamon Oil Soft Capsule in Patients with Functional Dyspepsia: A Randomized Double-Blind Placebo-Controlled Clinical Trial

**DOI:** 10.1155/2021/6634115

**Published:** 2021-05-13

**Authors:** Mehdi Zobeiri, Fatemeh Parvizi, Zahra Shahpiri, Fatemeh Heydarpour, Morteza Pourfarzam, Mohammad Reza Memarzadeh, Roja Rahimi, Mohammad Hosein Farzaei

**Affiliations:** ^1^Internal Medicine Department, Imam Reza Hospital, Kermanshah University of Medical Sciences, Kermanshah, Iran; ^2^Pharmaceutical Sciences Research Center, Kermanshah University of Medical Sciences, Kermanshah, Iran; ^3^Department of Traditional Pharmacy, School of Persian Medicine, Tehran University of Medical Sciences, Tehran, Iran; ^4^Social Development and Health Promotion Research Center, Health Institute, Kermanshah University of Medical Sciences, Kermanshah, Iran; ^5^Department of Clinical Biochemistry, School of Pharmacy and Pharmaceutical Sciences, Isfahan University of Medical Sciences, Isfahan, Iran; ^6^Medicinal Plant Research Center of Barij, Kashan, Iran

## Abstract

**Background:**

Different effects of cinnamon and its oil in traditional medicine in the treatment of diseases, including gastrointestinal diseases, were reported. The aim of this study is to evaluate the efficacy and safety of cinnamon oil (*Cinnamomum zeylanicum*) in patients with functional dyspepsia in a double-blind, randomized placebo-controlled trial.

**Methods:**

Soft gelatin capsule was made using the rotary die process, and the final capsule was standardized based on its cinnamaldehyde amount and analyzed by high-performance liquid chromatography (HPLC) method. Sixty-four patients with symptomatic functional dyspepsia were randomized to receive cinnamon oil soft capsule (*n* = 29) or sesame oil soft capsule as placebo (*n* = 35) for 6 weeks. The primary efficacy variable was the sum score of the patient's gastrointestinal symptom (five‐point scale). Secondary variables were the scores of each dyspeptic symptom including severity of vomiting, sickness, nausea, bloating, abdominal cramps, early satiety, acidic eructation/heartburn, loss of appetite, retrosternal discomfort, and epigastric pain/upper abdominal pain, as well as any reported adverse events.

**Results:**

The results showed that, after 6 weeks of treatment, the cinnamon oil and placebo groups significantly decreased the total dyspepsia score compared to the baseline at the endpoint (*P* < 0.001). However, there was no significant difference between the cinnamon oil and placebo groups in terms of the baseline and endpoint values of the outcome variables (*P*=0.317 and *P*=0.174, respectively). Two patients in the cinnamon oil group complained of rashes, and three patients in the placebo group complained of nausea.

**Conclusion:**

This study showed significant improvements in gastrointestinal symptom score in both treatment and placebo groups. However, there was no significant difference between the cinnamon oil and sesame oil groups in terms of the baseline and endpoint values of the outcome variables. This study was registered as https://clinicaltrials.gov/ct2/show/IRCT20170802035460N2, 29 December 2017, in the Iranian Registry of Clinical Trials with https://www.IRCT.ir.

## 1. Background

Functional dyspepsia (FD), a common gastroduodenal disorder, is defined by individual symptoms. Symptoms of functional dyspepsia such as postprandial fullness, early satiety, epigastric burning, or epigastric pain are often related to meal, but not to the abdominal pain, and affect one in five people in the community [1–3]. Effective therapies for functional dyspepsia are limited although acid secretion inhibitors, H2 blockers, proton pump inhibitors, prokinetics, *H. pylori* eradication treatment, antidepressants, psychotherapy, and mirtazapine may provide some symptom relief in clinical practice [1–4]. As there is no satisfactory medication for the treatment of FD, and the control of symptoms is a more realistic end point, more effective therapies should be presented as the main goal of treatment to achieve fewer adverse effects than conventional medications [2, 4]. Traditional medicines in treating functional gastrointestinal disorders are valuable sources for new drug discovery [2].

Commercial cinnamon is the inner bark of the *Cinnamomum zeylanicum* tree belonging to the Lauraceae family. Cinnamon, a fragrant spice plant, is a native of Sri Lanka and is commonly known in the trade as Ceylon cinnamon or Sri Lankan cinnamon [5, 6]. Besides food applications, the *Cinnamomum zeylanicum* has also been used for some health benefits including antimicrobial, antioxidant, anticholesterolemic, antiviral, antidiabetic, antitumor, analgesic, and antigastric ulcer effects [5, 7]. Due to the different effects of cinnamon and its oil in traditional medicine in the treatment of diseases, including gastrointestinal diseases, and since there have been no studies on its beneficial effects on dyspepsia, placebo-controlled trials are clearly needed to substantiate the efficacy of cinnamon oil. For this reason, we aimed to evaluate the efficacy of cinnamon oil (*Cinnamomum zeylanicum*) in the treatment of patients with functional dyspepsia with respect to the intensity of dyspeptic symptoms [8, 9].

## 2. Methods

### 2.1. Participants

A double-blind, randomized placebo-controlled clinical trial was conducted between April 2018 and May 2019 at an outpatient special clinic of Kermanshah University of Medical Sciences. A total of 64 patients with symptomatic functional dyspepsia were included in the study, and the allocation ratio was almost 1 : 1. This study was carried out in accordance with the Declaration of Helsinki and the research Ethics Committee of the Kermanshah University of Medical Sciences. Our study adheres to CONSORT guidelines, and the protocol was approved by the Ethics Committee of the Kermanshah University of Medical Sciences, Iran. All subjects were made aware of the content of the study, and written informed consent was obtained from each patient. For inclusion in the study, participants were required to be older than 18 years and younger than 80 years and their diseases are confirmed by complete medical evaluation, as well as patients who sign the testimonials and cooperate during the study and patients who have not been treated with cinnamon oil soft capsule during the last month. Exclusion criteria included the following: patients with inflammatory bowel disease, pure gastro-esophageal reflux, peptic ulcer disease, or irritable bowel syndrome; patients with a history of gastrointestinal system surgery; and pregnant and breast-feeding women.

### 2.2. Preparation of the Materials

#### 2.2.1. Cinnamon Oil

The dried barks of cinnamon were procured from local herbal market (Isfahan, Iran, 2016) and identified by the botanist of Herbarium Center at Faculty of Pharmacy, Tehran University of Medical Sciences, Tehran, Iran (*Cinnamomum zeylanicum* Nees, Voucher No. PMP- 908).

To prepare the cinnamon oil, according to the instruction of traditional manuscript (Qarabadin-e-kabir: a well-known Persian pharmacopeia), 500 g of plant's bark coarse powder was soaked 40 days in 3.26 L sesame oil (Golkaran Co., Kashan, Iran) in a glass closed vessel and exposed to the sun during extraction. Traditional cinnamon oil was obtained by filtration of supernatant and kept in dark containers.

#### 2.2.2. Cinnamon Oil Soft Capsules

**Figure 1:**
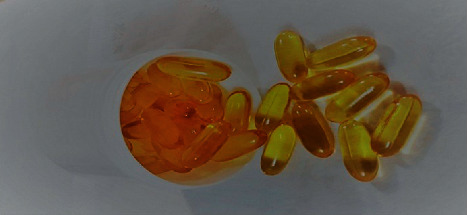
Cinnamon oil soft capsules.

Soft gelatin capsule was made using the rotary die process (Figure 1). In this formulation, traditional cinnamon oil was inserted into soft shell consist of gelatin, water, plasticizers, and preservative. The final capsule was standardized based on its cinnamaldehyde amount (55–75% of cinnamaldehyde as the significant pharmacological component of cinnamon essential oil) and analyzed by high-performance liquid chromatography (HPLC) method.

#### 2.2.3. HPLC Analysis of Cinnamon Oil

The cinnamaldehyde content of cinnamon oil was determined using a Shimadzu 10AD HPLC system (Kyoto, Japan) equipped with a column oven. Data integration was performed using Shimadzu Class-VP software. Separation was achieved using a Waters µBondpak C18 column (4.6 mm × 250 mm) and the column temperature was maintained at 30°C. The mobile phase comprising methanol (A) and 0.05% phosphoric acid (B) at a flow rate of 1.0 ml/min was used to elute the target components with a gradient program (0–5 min, 5%A; 5–10 min, 5%A to 35%A; 10–15 min, 35%A to 60%A; 15–18 min, 60%A to 80%A; 18–22 min, 80%A; 22–25 min, 80%A to 5%A, followed by 5 min equilibration between injections). The sample injection volume was 10 *µ*l and detection wavelength was set at 280 nm. The quantitation was by reference to a standard curve of cinnamaldehyde in sesame oil. Cinnamaldehyde was extracted from standards and samples using methanol in water by solvent extraction.

### 2.3. Study Design, Assessments, and Treatment

**Figure 2:**
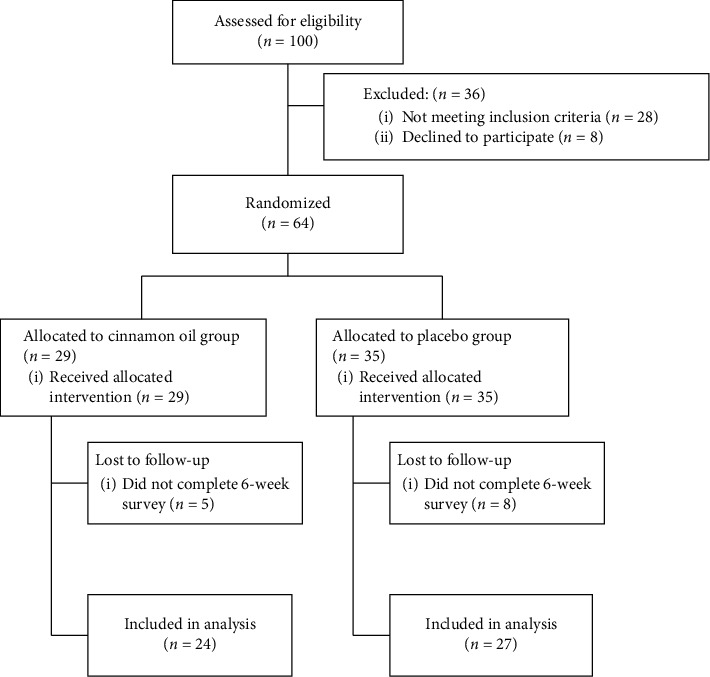
Flow chart of the trial.

After obtaining informed consent, eligible patients after colonoscopy were assessed at baseline for demographic characteristics (gender, age, height, weight, duration of functional dyspepsia, marital status, smoking, coffee drinker, tea drinker, regular meals, diet, stress, and previous drug-taking). They were then randomized to receive either cinnamon oil soft capsule (*n* = 29) or sesame oil soft capsule as placebo (*n* = 35). Sesame oil was purchased from Golkaran Agro-Industry Co., Kashan, Iran, obtained from Iranian white sesame seeds. Chemical composition analysis of this sesame oil showed that it contains 75 to 80% of liquid fatty acids (oleic and linoleic acids), 15% of solid fatty acids (palmitic, stearic, and arachidic acids), and 1% of lecithin. In this way, one soft capsule was used orally three times a day for a period of 6 weeks. For randomization procedure, we used a random number table. Odd number was allocated to an intervention group and even number was allocated to the placebo group. The capsule box of both groups looked identical, so in addition to the physicians and researchers, the patients were also blinded to the drug allocation.  Figure 2 shows a flow chart of the trial procedure.

To calculate the sample size, we first conducted a pilot study. Six patients who had functional dyspepsia were recruited and randomly assigned in two groups (placebo and cinnamon oil) for 6 weeks. The percent of bloating in two groups was 0% and 34% in cinnamon oil and placebo group. Using equation (1) (*α* = 0.05, *β* = 0.1) and taking into account 50% probability of falling in each group, the estimated minimum sample size was 32 and the total sample size was 64.

(1) $$\begin{array}{c} n=\frac{{\left(z_{1-\alpha /2}+z_{1-\beta }\right)}^{2}\left[P_{1}\left(1-P_{1}\right)+P_{2}\left(1-P_{2}\right)\right]}{{\left(P_{1}-P_{2}\right)}^{2}}≅21. \end{array}$$

### 2.4. Statistical Methods

Statistical analysis was performed using the Statistical Package for the Social Sciences (SPSS 16). Primary characteristics (age, height, weight, gender, marital status, duration of having functional dyspepsia, the status of being smoker, coffee drinker, and tea drinker and having regular meals, diet, stress, and previous drug-taking) and outcomes (gastrointestinal symptom score (GIS), as well as number of participants with any observed or reported adverse reaction) were compared between the patients in the cinnamon oil soft capsule and those in the placebo groups using the chi-square and Mann–Whitney tests, respectively. A Wilcoxon signed rank test was used for statistical comparison of values obtained before and after the intervention. All statistical tests were 2-sided, with the significance level set at 0.05.

## 3. Results

The HPLC analysis displayed that there was 2 ± 0.09 mg/g cinnamaldehyde in each cinnamon capsule. A total of 64 patients with symptomatic functional dyspepsia completed the clinical trial, 29 patients in treatment group and 35 patients in placebo group. The average age of patients was 39.7 years, at least 19 and at most 64 years. The patients in the treatment group included 10 males and 19 females. Also, the patients in the placebo group included 11 males and 24 females. The characteristics of the patients in both groups are listed in Table 1. There were no significant differences in the basic characteristics of the 2 groups (*P* > 0.05).

Patients were evaluated prior to and following 6 weeks in terms of the severity of vomiting, sickness, nausea, bloating, abdominal cramps, early satiety, acidic eructation/heartburn, loss of appetite, retrosternal discomfort, and epigastric pain/upper abdominal pain, as well as number of participants with any observed or reported adverse reaction. Symptom severity was assessed by a valid 5-point Likert scale: none (0), slight (1), moderate (2), severe (3), and very severe (4). The gastrointestinal symptom score (GIS) is used for the outcome measurement as a sum score, with its highest value of 40 points representing the most severe symptom intensity [10]. Based on the results, the GIS sum score showed nearly equal baseline values, with values of 20.72 and 21.94 for cinnamon oil and placebo groups, respectively (Table 2). In both groups, the GIS showed an improvement during 6 weeks (in the cinnamon oil group by 4.52 units, and in the placebo group by 7.19 units). The cinnamon oil and placebo groups significantly decreased the total dyspepsia score compared to the baseline at the endpoint (*P* < 0.001). However, there was no significant difference between the cinnamon oil and placebo groups in terms of the baseline and endpoint values of the outcome variables (*P*=0.317 and *P*=0.174, respectively) (Table 2). Moreover, the major symptoms analyzed in comparison of both groups are shown in Table 3. In all cases, the dyspepsia symptom score decrease compared to baseline was numerically greater in the placebo group than in the cinnamon oil group. As can be seen, the nausea score was decreased from baseline in the cinnamon oil group significantly compared to the placebo at the endpoint (*P*=0.012). However, the decreases of other dyspepsia symptoms scores from baseline in the cinnamon oil group were not significant compared to the placebo at the endpoint (*P* > 0.05) (Table 3).

Two patients in the cinnamon oil group complained of rashes, and three patients in the placebo group complained of nausea. No patient reported any other adverse events during the follow-up period in both groups.

## 4. Discussion

In this randomized double-blind placebo-controlled clinical trial, the efficacy and safety of cinnamon oil that has been claimed to be effective for treatment of patients with functional dyspepsia were assessed. To our knowledge, this study is the first evaluating cinnamon oil effects in functional dyspepsia. According to the potential pharmaceutical agent proven in several studies, it is possible to say that administration of this plant to the diet possibly attenuates the symptoms of gastrointestinal diseases.

Cinnamon, in addition to being a combination of antioxidants, anti-inflammatory, antimicrobes, antidiabetic, anticancer, hypoglycemic, and cardiovascular-reducing agents, has been reported to have beneficial effects on neurological disorders, including Parkinson's disease and Alzheimer's disease [11].

The protective effect of cinnamon ethanolic extract against carbon tetrachloride-induced liver injury in rats was investigated in 2012. Administration of ethanolic extract of cinnamon at different concentrations for 28 days can act as a potent hepatoprotective agent in poisoned rats, leading to a marked increase in the levels of catalase and superoxide dismutase enzymes and a decrease in the alanine aminotransferase, aspartate aminotransferase, and alkaline phosphatase [12]. Sahu et al. conducted a study to evaluate the skeletal muscle relaxant activity of aqueous extract of *Cinnamomum zeylanicum* compared to metocarbamol as a standard drug in white mice. Cinnamon aqueous extract showed better muscle relaxant effect than standard drug (metocarbamol, 60 mg/kg). This aqueous extract also showed fewer side effects compared to metocarbamol, which had less adverse effects attributed to its antioxidant properties [13]. Im et al. showed that polyphenolic content affects the antidiabetic activity and safety of cinnamon extract. Extracts that had increased 45 and 75% gallic acid equivalents of polyphenol content when administered to diabetic rat (200 mg per kg for 30 days) showed higher hypoglycemic and hypolipidemic effects than standard aqueous extract containing 15% gallic acid equivalents [14]. The results of Bharti et al.'s study showed that the antioxidant activity of cinnamon essential oil was higher than aqueous and alcoholic extracts. On the other hand, collagenase inhibitory activity in aqueous and alcoholic extracts was 25% and 30%, respectively, whereas in cinnamon essential oils it was maximum, 35%. Also, the antibacterial activity of cinnamon essential oil against all tested bacteria was significantly higher than the different cinnamon extracts [15].

Clinical studies on the efficacy of cinnamon oil have been mainly performed on blood pressure, blood glucose, lipid levels, and glycosylated hemoglobin levels, in patients with type 2 diabetes mellitus. The results showed that cinnamon oil is characterized by antidiabetic effects [16–19]. Ulrica von Arnim et al. performed a multicenter placebo-controlled double-blind study with 315 individuals to investigate the efficacy of herbal drug STW5 for patient with functional dyspepsia. Patients received 3 × 20 drops/day of STW5 or placebo, and a significant improvement was reported after 8 weeks by GIS score [20]. Clinical study of *Mentha pulegium* extract in fifty male and female patients was conducted to reduce symptoms of functional dyspepsia. 330 mg of extract was administered 3 times a day for 2 months in participants. They found that *Mentha pulegium* extract can significantly decrease total dyspepsia score, and some symptoms, including upper abdominal dull ache, bloating, belching, and stomach pain, compared to the placebo. Moreover, no significant change was observed in other symptom scores in the extract group [21].

## 5. Conclusion

This study showed significant improvements in gastrointestinal symptom score in both treatment and placebo groups. The cinnamon oil and placebo groups significantly decreased the total dyspepsia score compared to the baseline at the endpoint (*P* < 0.001). However, there was no significant difference between the cinnamon oil and placebo groups in terms of the baseline and endpoint values of the outcome variables (*P*=0.317 and *P*=0.174, respectively). Of course, for the condition, functional dyspepsia is subjective symptomatic condition which is heavily affected by placebo.

To our knowledge, this study is the first evaluating cinnamon oil effects in functional dyspepsia. However, the sample size was small and the experimental design was too simple without further analysis, which affects the reliability of this study. In addition, the short duration of patient follow-up was an important limitation of this study. Further studies are required to verify the efficacy and safety of the formula for symptom management on a larger scale, for a longer duration, and with different dosages.

## Figures and Tables

**Table 1:** Baseline characteristics at inclusion.

Characteristics		Treatment group *n* (%)	Placebo group *n* (%)	*P* value
Mean age (years)		41.9	37.63	0.2^*∗∗*^
Height (cm)		168.12	167.23	0.692^*∗∗*^
Weight (kg)		72.21	71.83	0.905^*∗∗*^
Duration of functional dyspepsia (months)		64.13	145	0.067^*∗∗∗*^

*Gender*				
Male		10 (34.5)	11 (31.4)	0.796
Female		19 (65.5)	24 (68.6)	

*Marital status*				
Single		10 (35.7)	9 (27.3)	0.478
Married		18 (64.3)	24 (72.7)	

*Smoking*				
No		26 (92.9)	33 (100)	0.207^*∗*^
Yes		2 (7.1)	0 (0)	

*Coffee drinker*				
No		26 (89.7)	32 (100)	0.102^*∗*^
Yes		3 (10.3)	0 (0)	

*Tea drinker*				
No		3 (10.3)	2 (5.9)	0.654^*∗*^
Yes		26 (89.7)	32 (94.1)	

*Regular meals*				
No		10 (35.7)	5 (15.2)	0.063
Yes		18 (64.3)	28 (84.8)	

*Diet*				
No		27 (96.4)	31 (93.9)	1^*∗*^
Yes		1 (3.6)	2 (6.1)	

*Stress*				
No		5 (17.2)	30 (90.9)	0.456^*∗*^
Yes		24 (82.8)	3 (9.1)	

*Previous drug-taking*				
No		6 (20.7)	6 (17.6)	0.759
Yes		23 (79.3)	28 (82.4)	

**Table 2:** The GIS index in the cinnamon oil and placebo groups before and after 6 weeks of intervention. The data are expressed as mean.

	Cinnamon oil	Placebo	Between-groups *P* value
Baseline score	20.72	21.94	0.317
After 6 weeks	16.2	14.75	0.174
Within-group *P* value	<0.001	<0.001	

**Table 3:** The dyspepsia symptom score decrease compared to baseline in the cinnamon oil and placebo groups. The data are expressed as mean.

	Cinnamon oil	Placebo	Between-groups *P* value
Vomiting	0.0000	0.0000	1
Sickness	0.1667	0.3077	0.733
Nausea	0.0000	0.4231	0.012
Bloating	1.0400	1.3929	0.191
Abdominal cramps	0 .7200	0.8462	0.804
Early satiety	0.1250	0.5000	0.059
Acidic eructation/heartburn	0.1905	0.5000	0.214
Loss of appetite	0.0833	0.4167	0.252
Retrosternal discomfort	0.1250	0.2963	0.481
Epigastric pain/upper abdominal pain	0.4348	0.6154	0.69

## Data Availability

The numerical data used to support the findings of this study are included within the article, and the rough data are available from the corresponding author upon request.

## Abbreviations

FD: Functional dyspepsia
HPLC: High-performance liquid chromatography
GIS: Gastrointestinal Symptom Score.
